# Supplementary material for: HFR1, a bHLH Transcriptional Regulator from Arabidopsis thaliana, Improves Grain Yield, Shade and Osmotic Stress Tolerances in Common Wheat
Source: Int J Mol Sci. 2022 Oct 10;23(19):12057. doi: 10.3390/ijms231912057 (PMC9569703; doi:10.3390/ijms231912057)
Supplement: Supplementary file 1 [file ijms-23-12057-s001.zip › ijms-1902676-supplementary.pdf]

## Supplementary Materials

**Figure S1.** Identification of the *AtHFR1* transgenic lines and morphology of non-transgenic cv. Fielder (WT) and *AtHFR1* transgenic lines under different light-conditions, high-R/FR, low-R/FR, high-B or low-B conditions.

**Figure S2.** Relative expression level of *AtHFR1* in transgenic wheat lines.

**Figure S3.** Relative expression levels of *TaFT1*, *TaCO1* and *TaCO2.*

**Figure S4.** Grain length and width of the WT (cv. Fielder) and *AtHFR1* transgenic lines.

**Figure S5.** Morphology and chlorophyll contents of the WT (cv. Fielder) and *AtHFR1* transgenic lines (#498 and #511) under long-day condition for 20 days.

**Table S1.** Internode lengths of *AtHFR1* transgenic lines.

**Table S2.** Agronomic traits of *AtHFR1* transgenic lines.

**Table S3.** List of primers used in this study.

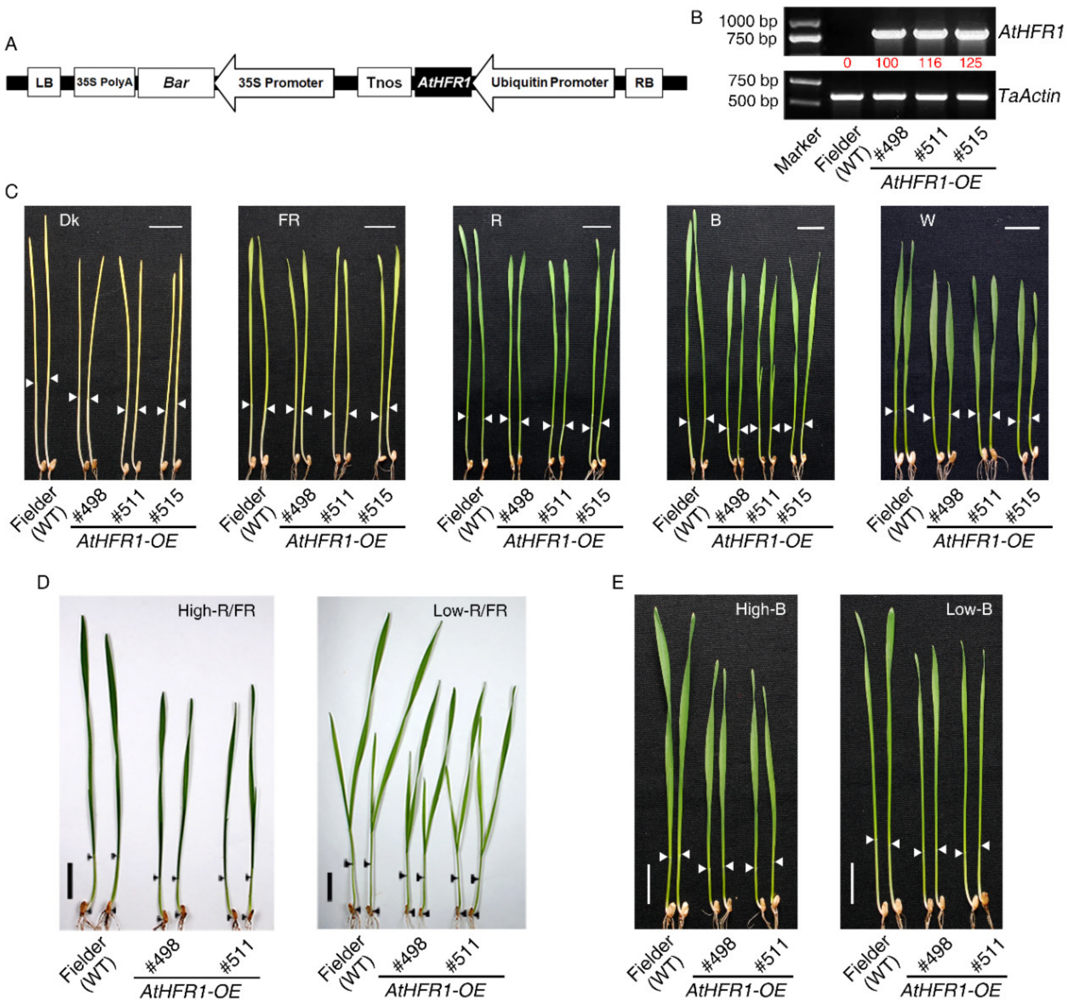


**Figure S1. Identification of the *AtHFR1* transgenic lines and morphology of non-transgenic cv. Fielder (WT) and *AtHFR1* transgenic lines under different light-conditions, high-R/FR, low-R/FR, high-B or low-B conditions.**

(A) Information of recombinant plasmid *pBCXUN-AtHFR1*. (B) Semi-RT-PCR analysis for *AtHFR1* expression in the WT and *AtHFR1* transgenic lines (#498, #511, and #515). Numbers under lanes indicate the relative band intensities to quantify *Actin* and are normalized for each panel. (C) Morphology of the WT and the *AtHFR1* transgenic lines (#498, #511, and #515). Seedlings were grown under dark (Dk), far-red (FR, 10 μmol·m$^{-2}$·s$^{-1}$), red (R, 15 μmol·m$^{-2}$·s$^{-1}$), blue (B, 15 μmol·m$^{-2}$·s$^{-1}$) or white (W, 100 μmol·m$^{-2}$·s$^{-1}$) light condition for 7 d. Bar = 2 cm. (D) Morphology of the Fielder and the *AtHFR1* transgenic lines (#498 and #511) under high-R/FR (R, 96 μmol·m$^{-2}$·s$^{-1}$; FR, 21 μmol·m$^{-2}$·s$^{-1}$; B, 15 μmol·m$^{-2}$·s$^{-1}$) or low-R/FR (R 12 μmol·m$^{-2}$·s$^{-1}$; FR 105 μmol·m$^{-2}$·s$^{-1}$; B 15 μmol·m$^{-2}$·s$^{-1}$) for 7 d. Bar = 2 cm. (E) Morphology of the Fielder and the *AtHFR1* transgenic lines (#498 and #511) under high-B (50 μmol·m$^{-2}$·s$^{-1}$) or low-B (2 μmol·m$^{-2}$·s$^{-1}$) for 7 d. Bar = 2 cm. Triangles in (C), (D) and (E) are showing the positions of coleoptile.

Related to Figure 1.

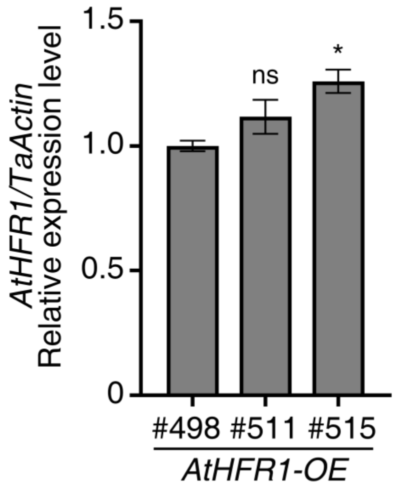


**Figure S2. Relative expression levels of *AtHFR1* in transgenic wheat lines.**

Seedlings grown under long-day condition for 8 days were used to analyze *AtHFR1* expression level. Data are means ± SEs, n = 3. ns indicates no significant difference and asterisks denote significant differences of #498, #511, or #515 with the WT according to ANOVA (* $P < 0.05$; ** $P < 0.01$).

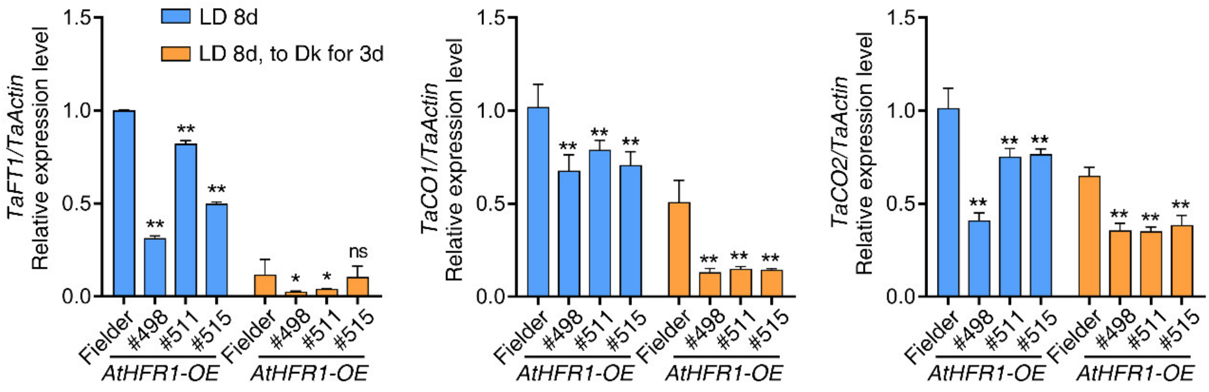


**Figure S3. Relative expression levels of *TaFT1*, *TaCO1* and *TaCO2.***

Seedlings were grown under long-day conditions for 8 days, and then transferred to the dark for 3 days to eliminate the effect of photoperiod on gene expression. Data are means ± SEs, n = 3. Asterisks denote significant differences of #498, #511, or #515 with the WT according to ANOVA (ns indicates no significant difference; * $P < 0.05$; ** $P < 0.01$).

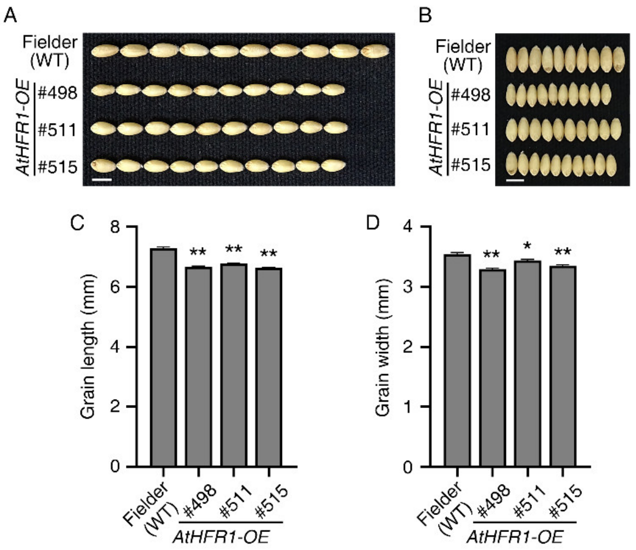


**Figure S4. Grain length and width of the WT (cv. Fielder) and *AtHFR1* transgenic lines.**

Grain morphology showing grain length (A) and grain width (B) and quantification of grain length (C) and grain width (D) of the WT and *AtHFR1* transgenic lines (#498, #511, and #515). Data are means ± SEs, n = 15. Asterisks denote significant differences of #498, #511, or #515 with the WT according to ANOVA (* $P < 0.05$; ** $P < 0.01$).

Related to Figure 4.

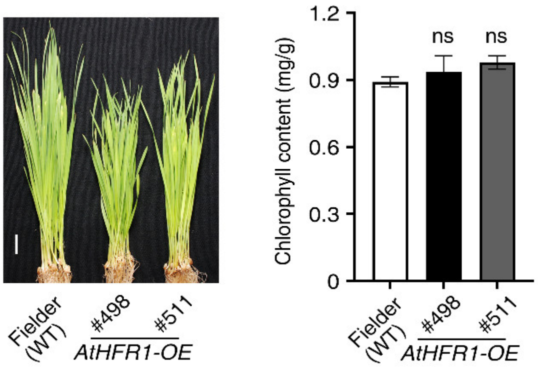


**Figure S5. Morphology and chlorophyll contents of the WT (cv. Fielder) and *AtHFR1* transgenic lines (#498 and #511) under long-day condition for 20 days.**

Data are means ± SEs, n ⩾ 5. ns indicates no significant difference of #498 or #511 with Fielder according to ANOVA.

Related to Figure 6.

**Table S1. Internode lengths (cm) of *AtHFR1* transgenic lines.**

| | | Fielder (WT) | *AtHFR1-OE* | | |
|---|---|---|---|---|---|
| | | | #498 | #511 | #515 |
| Internode (top to bottom) | spike | 13.1 ± 1.28 | 12.07 ± 0.76 * | 12.1 ± 1.25 * | 13.11 ± 1.21 |
| | 1st | 37.15 ± 4.96 | 27.14 ± 1.88 ** | 26.43 ± 3.46 ** | 28.79 ± 4.32 ** |
| | 2nd | 25.16 ± 1.68 | 19.26 ± 1.26 ** | 20.52 ± 1.25 ** | 20.53 ± 3.03 ** |
| | 3rd | 19.3 ± 2.47 | 14.48 ± 1.54 ** | 15.71 ± 2.43 ** | 16.78 ± 2.22 ** |
| | 4th | 13.3 ± 2.81 | 10.1 ± 1.15 ** | 9.38 ± 3.03 ** | 10.63 ± 1.8 ** |
| | 5th | 8.62 ± 3.64 | 4.37 ± 1.42 ** | 3.82 ± 1.82 ** | 4.16 ± 1.34 ** |

Materials were planted in field (near Zhengzhou, ≈34.9° N, ≈113.6° E) from Oct. 2019 to May 2020. After harvest, Internode lengths were measured. Data are means ± SDs, Asterisks denote significant differences of #498, #511, or #515 with the WT according to ANOVA (* $P$ < 0.05; ** $P$ < 0.01).

**Table S2. Agronomic traits of *AtHFR1* transgenic lines.**

| | Fielder (WT) | *AtHFR1-OE* | | |
|---|---|---|---|---|
| | | #498 | #511 | #515 |
| Plant height (cm) | 116 ± 4.87 | 87.66 ± 2.30 ** | 87.11 ± 3.86 ** | 93.24 ± 3.96 ** |
| Spikes per plant | 22.45 ± 6.81 | 35.67 ± 10.36 ** | 34.41 ± 7.66 ** | 34.28 ± 7.85 ** |
| Heading data (d) | 181.1 ± 1.78 | 192.2 ± 0.73 ** | 190.8 ± 1.24 ** | 189.2 ± 1.49 ** |
| Thousand-gain weight (g) | 42.88 ± 4.96 | 30.27 ± 2.38 ** | 36.58 ± 2.50 * | 35.91 ± 3.16 * |
| Grain number per spike | 64.41 ± 6.37 | 59.59±3.89 * | 61.35 ± 3.42 | 60.76 ± 5.77 |
| Grain yield per plant (g) | 39.23 ± 3.61 | 46.92 ± 4.17 ** | 49.57 ± 7.12 * | 47.84 ± 6.0 * |
| Grain length (mm) | 7.381 ± 0.08 | 6.45 ± 0.1 ** | 6.45 ± 0.06 ** | 6.357 ± 0.11 ** |
| Grain width (mm) | 3.45 ± 0.11 | 3.186 ± 0.04 ** | 3.286 ± 0.06 * | 3.243 ± 0.05 ** |
| Length of flag leaf (mm) | 235.2 ± 12.95 | 156.6 ± 8.43 ** | 160.3 ± 7.04 ** | 163.4 ± 6.49 ** |
| Width of flag leaf (mm) | 22.06 ± 1.12 | 17.69 ± 0.6 ** | 16.5 ± 0.63 ** | 15.19 ± 0.75 ** |

Materials were planted in field (near Zhengzhou, ≈34.9° N, ≈113.6° E) from Oct. 2019 to May 2020. The agronomic traits were investigated according to the method described in 4.3. Data are means ± SDs. Asterisks denote significant differences of #498, #511, or #515 with the WT according to ANOVA (* $P < 0.05$; ** $P < 0.01$).

**Table S3. List of primers used in this study**

| Gene | Primer | Sequence (5'→3') | Use | Reference |
|---|---|---|---|---|
| *AtHFR1* (At1g02340) | AtHFR1-F1 | ATGTCGAATAATCAAGCTTTC | Gene cloning and vector construction | |
| | AtHFR1-R1 | TCATAGTCTTCTCATCGCATG | | |
| | AtHFR1-828R | AGGAACCAAACCGTGAAG | RT-PCR identification of transgenic lines | |
| | AtHFR1-28F | TTGGGATGGAGAAACGAC | | |
| *TaACTIN* (AB181991.1) | TaAct-F2 | CTATCCTTCGTTTGGACCTTG | As an internal control of RT-PCR | |
| | TaAct-R2 | CGGGACCAGACTCATCGT | | |
| | Ta-Act F | TACTCCCTCACAACAACC | As an internal control of real-time PCR | |
| | Ta-Act R | GCTCCTGCTCATAATCAAG | | |
| *TaCAB* (XM_044587543.1) | TaCAB-QF1 | AAGGTGAAGGAAATCAAGAAC | Marker gene for photomorphogenesis | |
| | TaCAB-QR1 | ACCCTTACCAGTGACGAT | | |
| *TaCHS* (XM_044598705.1, XM_044526325.1) | TaCHS-QF1 | ACTACTACTTCAGGGTCACC | Marker gene for photomorphogenesis | |
| | TaCHS-QR1 | ACTTCTCGCACATCCTCT | | |
| *TaFT1* (DQ890162.1) | TaFT-F3 | CAGCAGCCCAGGGTTGAG | Marker genes for heading | Yan et al., 2006 |
| | TaFT-R3 | ATCTGGGTCTACCATCACGAGTG | | |
| *TaCO1* | CO1-AB-F3 | CACATCAGAGTGGTTATGC | Marker genes for heading | Chen et al., 2014 |
| | CO1-AB-R3 | GGACTGGACCGTATTGTC | | |
| *TaCO2* | CO2-AB-SYB-F4 | AAGGGTGTGAGTGTGTAG | Marker genes for heading | Chen et al., 2014 |
| | CO2-AB-SYB-R4 | GATATGTCATTGCTGATGGAAG | | |
| *TaSGR* (XM_044526410.1, XM_044533918.1, XM_044542480.1) | TaSGR-QF | CGTCCACTGCCACATCTCCG | Real-time PCR | |
| | TaSGR-QF | CACGAACGCCTTCAGAACCAC | | |

| | | | |
|---|---|---|---|
| *TaPIL13* (XM_044530914.1, XM_044530915.1) | PIL13-QF | GACCTCTTCTCGGAGATC | Real-time PCR |
| | PIL13-QR | TTCGCTGCTTATCATCCT | |
| *TaPIL15-1B* (XM_044531643.1, XM_044531644.1) | PIL15-1B-QF | GTCGGAGAGGAGGAGAAG | Real-time PCR |
| | PIL15-1B-QR | ACGCCTTGTCAATCTTGT | |
| *TaPIL15-1D* (XM_044592408.1, XM_044592409.1) | PIL15-1D-QF | GAGAAGGGACCGAATCAA | Real-time PCR |
| | PIL15-1D-QR | GCCTTGTCGATCTTGTTG | |

Reference


Yan, L., Fu, D., Li, C., Blechl, A., Tranquilli, G., Bonafede, M., Sanchez, A., Valarik, M., Yasuda, S. and Dubcovsky, J. (2006) The wheat and barley vernalization gene *VRN3* is an orthologue of *FT*. *Proc. Natl. Acad. Sci. USA*, **103**, 19581-19586.

Chen, A., Li, C., Hu, W., Lau, M.Y., Lin, H., Rockwell, N.C., Martin, S.S., Jernstedt, J.A., Lagarias, J.C. and Dubcovsky, J. (2014) Phytochrome C plays a major role in the acceleration of wheat flowering under long-day photoperiod. *Proc. Natl. Acad. Sci. USA*, **111**, 10037-1004
